# Down-regulated m6A reader FTO destabilizes PHF1 that triggers enhanced stemness capacity and tumor progression in lung adenocarcinoma

**DOI:** 10.1038/s41420-022-01125-y

**Published:** 2022-08-09

**Authors:** Jinfeng Ning, Fengjiao Wang, Jianlong Bu, Kaibin Zhu, Wei Liu

**Affiliations:** 1 Department of Thoracic Surgery, Harbin Medical University Cancer Hospital, No. 150, Haping Road, Harbin, 150081 Heilongjiang China; 2 The forth department of medical oncology, Harbin Medical University Cancer Hospital, No. 150, Haping Road, Harbin, 150081 Heilongjiang China

**Keywords:** Non-small-cell lung cancer, Chromatin remodelling

## Abstract

Aberrant epigenetic drivers or suppressors contribute to LUAD progression and drug resistance, including KRAS, PTEN, Keap1. Human Plant Homeodomain (PHD) finger protein 1 (PHF1) coordinates with H3K36me3 to increase nucleosomal DNA accessibility. Previous studies revealed that PHF1 is markedly upregulated in various tumors and enhances cell proliferation, migration and tumorigenesis. However, its roles in LUAD are still unknown. We aimed to depict the biological roles of PHF1 and identify useful targets for clinical treatment of LUAD. Based on the bioinformatic analysis, we found that PHF1 was down-regulated in LUAD samples and low PHF1 expressions correlated with unfavorable clinical characteristics. Patients with low PHF1 had poorer survival outcomes relative to those with high PHF1. Targeting PHF1 potentiated cell growth, migration and in vivo proliferation. Mechanistically, FTO mediated the stabilization of PHF1 mRNA by demethylating m6A, which particularly prevented YTHDF2 from degrading PHF1 transcripts. Of note, FTO also expressed lowly in LUAD that predicts poor prognosis of patients. FTO inhibition promoted LUAD progression, and PHF1 overexpression could reverse the effect. Lastly, down-regulated FTO/PHF1 axis could mainly elevate FOXM1 expression to potentiate the self-renewal capacity. Targeting FOXM1 was effective to suppress PHF1^low/−^ LUAD growth. Collectively, our findings revealed that FTO positively regulates PHF1 expression and determined the tumor-suppressive role of FTO/PHF1 axis, thereby highlighting insights into its epigenetic remodeling mechanisms in LUAD progression and treatment.

## Background

Lung cancer is the leading cause of cancer-related mortality worldwide, leading to more than 1 million deaths every year [1]. Accounting for nearly 45% of lung cancer patients, lung adenocarcinoma (LUAD) is the most common subtype, followed by lung squamous cell carcinoma [2]. Although remarkable improvements in LUAD diagnosis and treatment have been obtained, including surgical resection, targeted drugs and immunological therapies, the prognosis of LUAD still remains poor and the 5-year survival rate varies from ~10 to ~15% [3, 4]. The main reasons of mortality in LUAD patients contain delayed diagnosis, distal metastasis, as well as drug resistance. During the past decades, intensive efforts, like high-throughput sequencing researches, have identified main oncogenic drivers in LUAD tumorigenesis, including KRAS, EGFR, FGFR4, VEGFA, e.g., [5, 6]. However, there is an urgent need to find novel therapeutic targets due to tumor heterogeneity, improving the understanding of biological and molecular processes in LUAD.

As reported, genetic and epigenetic alterations both participate in the multiple processes of tumorigenesis [7, 8]. Epigenetics is an essential regulatory mechanism of gene expression that contains DNA methylation, histone modification, noncoding RNA regulation, and chromatin remodeling formation [9]. Aberrant epigenetic regulations are regarded to play important roles in tumor initiation, metastasis, and treatment resistance [10]. As the hotspot of epigenetic research, the crucial roles of N6-methyladenosine (m6A) were demonstrated to participate in various malignancies, including LUAD [11]. As the most important internal posttranscriptional methylation of mRNAs, m6A is a reversible process regulated by the m6A system containing writers (W), erasers (E), and readers (R). The eraser, like FTO or ALKBH5, is responsible for catalyzing demethylation, whereas major m6A writers, like METTL3/14, WTAP, or KIAA1429 play the methyltransferase roles. As reported, FTO is the first m6A demethylase to exert effects on mRNA in an iron-dependent manner. Previous studies have found that FTO was related to increased body mass and obesity [12]. FTO deletion could result in lower body weight and attenuate anxiety-related behaviors. FTO-KO mice were more resistant to stress stimulation, suggesting the vital function of FTO in pathogenesis of depression [13]. In recent years, intensive studies have uncovered the potential associations between FTO and tumorigenesis. As indicated, FTO mediated the m6A demethylation in the 3′UTR of BNIP3 mRNA to enhance its degradation, driving tumorigenesis of breast cancer [14]. In multiple myeloma, IDH2-mediated FTO activation could reduce the m6A level on WNT7B transcripts, thereby elevating WNT7B expression and thus activating Wnt signaling crosstalk [15]. In contrast, a novel tumor suppressor role of the RNA demethylase FTO indicates m6A RNA modifications in the regulation of cyclic AMP signaling involved in stemness and tumor initiation. FTO could inhibit the self-renewal of ovarian cell stem cells (CSCs) and suppress in vivo tumorigenesis [16]. Besides, Zhang et al. investigated that activation of FTO induced by an increased intracellular α-ketoglutarate–to-succinate ratio could stabilize BRD9 to assemble super enhancers to drive tumor growth of HIF2α^low/−^ ccRCC [17]. However, FTO was not indispensable for the growth of HIF2α^high^ ccRCC, indicating the different roles of FTO in distinct genetic backgrounds of one tumor. In addition, FTO upregulated the expression of E2F1 by inhibiting the m6A modification of E2F1 to augment the viability, migration, and invasion of NSCLC cells [18]. Conversely, EZH2 was reported to enhance H3K27me3 and inhibit FTO expression in LUAD, indicating that FTO is a tumor suppressor in LUAD. Therefore, the specific roles of FTO in LUAD progression remain indefinite and controversial.

The Polycomb repressive complex 2 (PRC2) is considered to be a multicomponent histone H3K27 methyltransferase complex, which is mainly responsible for silencing the Hox genes during embryonic development [19]. Among these components, the Polycomb-like proteins PHF1, MTF2, and PHF19 are essential for maintaining the catalytic activity of PRC2 [20]. Of note, the Tudor domain of human PHF1 binds to and recognizes histone H3 trimethylated at Lys36 (H3K36me3) [21]. Besides, the N-terminal PHD finger of PHF1 could also recognize symmetric dimethylation of H4R3 (H4R3me2s) catalyzed by PRMT5-WDR77, indicating the multiple roles of PHF1 in epigenetic regulation and genome maintenance. Previous studies have shown that PHF1 fusions induce specific gene expression and chromatin accessibility profiles in ossifying fibromyxoid tumors and mesenchymal cells [22]. Moreover, PHF1 promotes cell proliferation, invasion, and tumorigenesis in vivo and in vitro and its expression is markedly elevated in a variety of human cancers [23]. Nevertheless, so far, no relevant research are available to elucidate the associations between PHF1 and pathogenesis of LUAD.

In this study, we found that PHF1 expressed lowly in LUAD, which was a tumor suppressor. FTO mediated the demethylation m6A of PHF1 to sustain its mRNA levels. We further uncovered the relationships between FTO/PHF1 axis and self-renewal of LUAD CSCs, providing novel insights and therapeutical targets in LUAD treatment.

## Results

### Identification of PHF1 as an essential epigenetic suppressor in LUAD

**Fig. 1:**
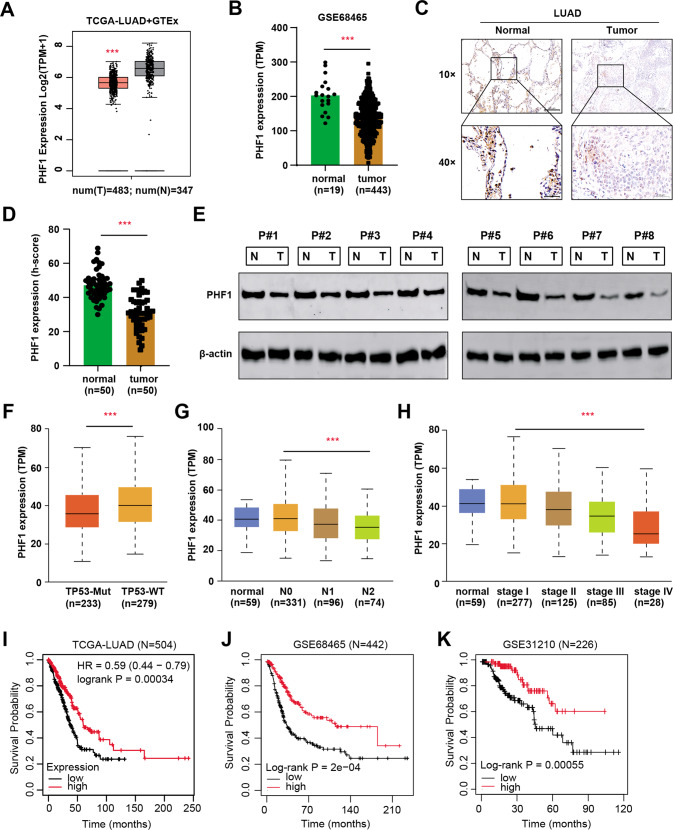
PHF1 is down-regulated in LUAD that predicts poor prognosis. **A** Box plot of PHF1 mRNA levels in normal (T = 483) and tumor samples (*N* = 347) based on the TCGA-LUAD and GTEx-Lung expression data. **B** Differential analysis of PHF1 mRNA levels in normal and tumor tissues based on the expression data of GSE68465 dataset. **C** Representative immunohistochemical (IHC) graphs of PHF1 levels in human normal and LUAD samples. Upper scale = 200 μm, Lower scale = 50 μm. **D** Quantification of PHF1 h-scores in normal and tumor samples showing that PHF1 levels were notably down-regulated in LUAD samples. **E** Western-blotting assay detecting the PHF1 proteins in paired tissues from eight patients with LUAD. **F–H** Correlation analysis showed that lower PHF1 expression levels were associated with TP53 mutaion (**F**), lymphatic metastasis (**G**), and clinicalpathological stages (**H**). **I–K** Kaplan–Meier analysis in three independent datasets showed that patients with low PHF1 suffered from poorer prognosis relative to those with high PHF1 levels, including TCGA-LUAD cohort (**I**, log-rank *P* < 0.001, *N* = 504), GSE68465 dataset (**J** log-rank *P* < 0.001, *N* = 442) and GSE31210 dataset (**K** log-rank *P* < 0.001, *N* = 226). Bar = Mean ± SD. *P < 0.05, **P < 0.01, ***P < 0.001.

Intensive evidence has indicated that PHF1 plays an essential role in the pathogenesis of tumors, but its functions in LUAD have never been reported. To determine whether PHF1 is an epigenetic regulator with clinical significance, we firstly conducted the differential analysis in TCGA-LUAD (*N* = 483) and GSE68465 (*N* = 443) datasets, where PHF1 was found to be significantly lower in tumor samples than normal tissues (Fig. 1A–B). The clinical data was summarized in Table S1. To validate the bioinformatic analysis, we collected totally 50 LUAD samples with paired normal tissue and conducted the immunohistochemical (IHC) staining assay. The protein levels of PHF1 were significantly lower versus those in paired normal tissues, as indicated by quantification of PHF1 h-scores (Fig. 1C–D). Meanwhile, eight paired fresh LUAD and normal samples were used to detect PHF1 proteins and western blotting assay revealed that PHF1 proteins were remarkably down-regulated in LUAD samples (Fig. 1E). Subsequently, we re-analyzed the PHF1 mRNA data in TCGA-LUAD cohort and correlation analysis showed that PHF1 levels were negatively related to TP-53 mutation type, N stages, as well as clinicalpathological stages (Fig. 1F–H). Last of all, we also analyzed the PHF1 levels with survival information from three databases, like TCGA-LUAD, GSE68465 and GSE31210, to further confirm whether PHF1 is a prognostic marker for LUAD. Kaplan–Meier analysis showed that patients with low PHF1 expressions underwent poorer overall survival (OS) outcomes as compared to those with high PHF1 levels, which exhibited the consistent results in three independent cohorts (Fig. 1I–K). Taken together, these data implicated that PHF1 expressed lowly in LUAD samples and low PHF1 levels predicted poor prognosis for LUAD patients.

### Targeting PHF1 enhanced LUAD cells proliferation and migration in vitro and in vivo

**Fig. 2:**
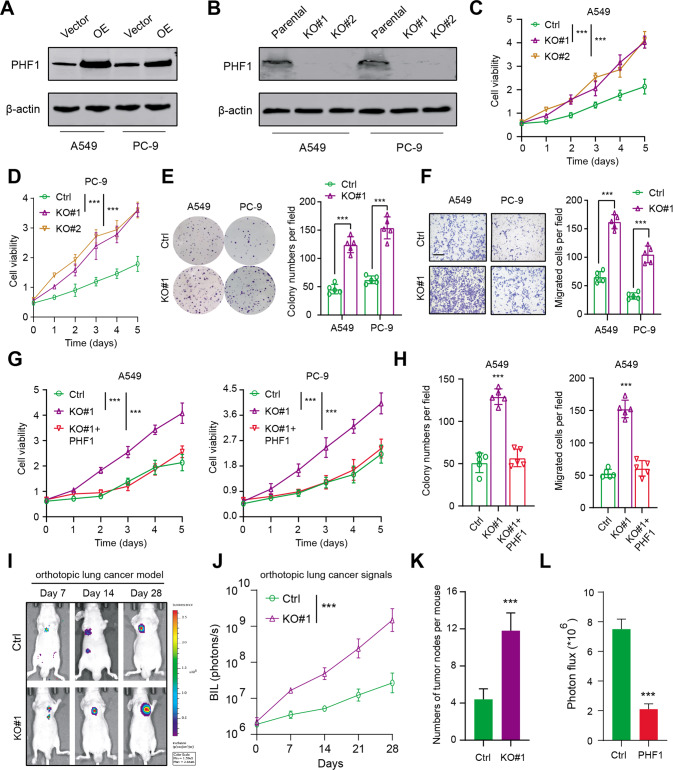
Experimental assays demonstrated that PHF1 functions as a biological tumor supperessor in LUAD. **A** Western blotting showing the ectopic expression of PHF1 in A549 and PC-9 cells. **B** Western blotting showing the *CRISPR/Cas9*-mediated KO of PHF1 in LUAD cells. **C** MTT assay was conducted to assess the effect of PHF1 deficiency on A549 cell viability. **D** MTT assay was conducted to assess the effect of PHF1 deficiency on PC-9 cell viability. **E** Colony formation assay revealing the effect of PHF1-KO on cell clone growth capacity (A549 and PC-9). **F** Cell migration assays showing the effect of PHF1-KO on migratory abilities of LUAD cells (A549, PC-9). **G** PHF1 deficiency notably enhanced the proliferation of A549 and PC-9 cells, wheras ectopic expression of PHF1 restored the in vitro growth of cells. **H** Colony formation assay and migration assay were performd in three cell groups (Ctrl, KO#1, KO#1 + PHF1) to determine whether PHF1 overexpression could restore the malignant features of cells. **I** Representative bioluminescence (BIL) imaging graphs showing the in vivo orthotopic tumor growth in two groups. **J** Quantification of orthotopic lung tumor signals in two groups which were detected at the indicated timepoints. **K** Quantification of orthotopic tumor leision numbers via dissecting the mice lung in the indicated groups. **L** Quantification of BIL signals in the Ctrl and PH1-OE groups at the Day 28. Bar = Mean ± SD. *P < 0.05, **P < 0.01, ***P < 0.001.

To investigate the biological roles of PHF1 in LUAD cell malignant features, we utilized the gene editing technology to delete PHF1 in A549 and PC-9 cells, which were confirmed by western blotting (Fig. 2A). Besides, we also constructed PHF1-overexpressing LUAD cells (Fig. 2B). The 3-[4,5-dimethylthiazol-2-yl]−2,5 diphenyl tetrazolium bromide (MTT) assays indicated that PHF1 deficiency indeed resulted in a significant increase in growth curve of A549 and PC-9 cells (Fig. 2C–D). Then, the colony formation assay also demonstrated that PHF1 ablation could promote cell growth in vitro compared with parental cells (Fig. 2E). Moreover, the migration ability of A549 and PC-9 cells was also potentiated when PHF1 was deleted (Fig. 2F). Notably, ectopic expression of PHF1 in PHF1-KO cells could suppress and restore the cell growth and migration capacity (Fig. 2G–H). To further evaluate the in vivo effect of PHF1 on regulating cell growth, we utilized the PHF1-deficient A549 cells and parental cells to construct the orthotopic lung cancer model. Expectedly, after 8 weeks, PHF1 deficiency remarkably enhanced growth of orthotopic lung tumors, as indicated by the bioluminescence signals and numbers of tumor nodes (Fig. 2I–K). In contrast, orthotopic tumors derived from PC-9-luciferase cells overexpressing PHF1 significantly delayed versus thoses derived from corresponding control cells (Fig. 2L).

### FTO stabilizes PHF1 mRNA by demethylating m6A

**Fig. 3:**
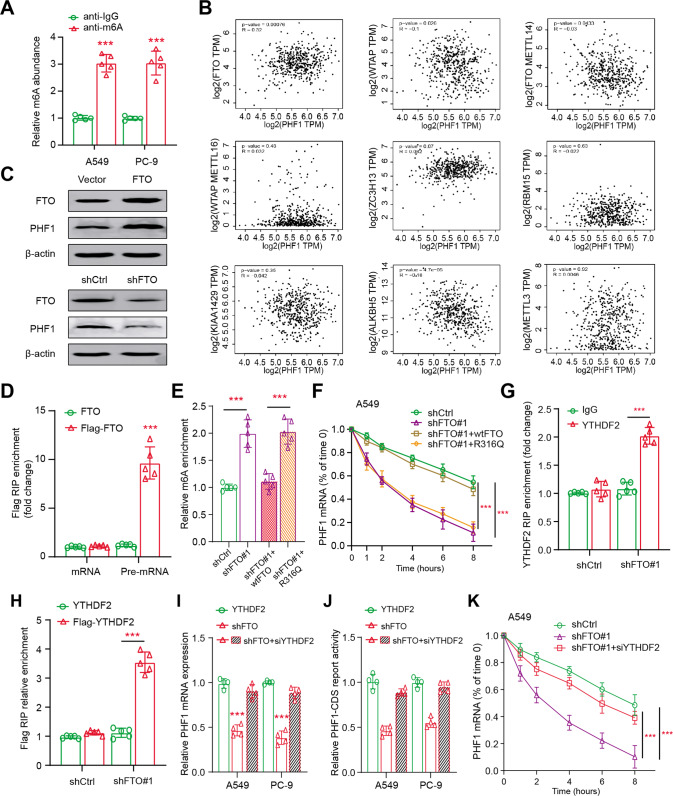
FTO mediates the stability of PHF1 mRNA by demethylating m6A process. **A** MeRIP assay was used to detect the m6A abundance on PHF1 mRNA in A549 and PC-9 cells. **B** Gene correlation analysis of PHF1 and m6A-related enzymes using Spearman statistics in TCGA-LUAD cohort. **C** Western blotting assay showing the protein levels and associations of FTO and PHF1 in LUAD cells. **D** RIP assay showing the endogenous interaction between FTO and PHF1 pre-mRNA using the nuclear extracts of A549 cells expressing FTO with or without Flag tag. **E** MeRIP-qPCR analysis indicating the m6A modification of PHF1 pre-mRNA in FTO-KD cells with or without WT or R316Q mutant expression. **F** FTO-KD A549 cells with or without WT or R316Q mutant expression were treated by actinomycin D at the different timepoints. The levels of remaining PHF1 mRNA were determined via qPCR assay. **G** RIP assay showing the interaction between endogenous YTHDF2 and PHF1 pre-mRNA in A549 cells with or without FTO-KD. **H** RIP analysis of transcripts from nuclear extracts of control and FTO-KD A549 cells expressing YTHDF2 or Flag-YTHDF2. **I** The qPCR assays detecting the PHF1 mRNA levels in FTO-KD A549 and PC-9 cells treated with or without YTHDF2 siRNA. **J** The PHF1 3′UTR firefly luciferase activity was detected in FTO-KD A549 and PC-9 cells treated with or without YTHDF2 siRNA. **K** FTO-KD A549 cells treated with or without siYTHDF2 were cultured with actinomycin D at the indicated timepoints. The qPCR assays were performed to detect the amount of remaining PHF1 levels after the above treatment. Bar = Mean ± SD. *P < 0.05, **P < 0.01, ***P < 0.001.

To explain the function of m6A methylation in the epigenetic regulation of PHF1, we determined whether PHF1 mRNA could be efficiently m6A-methylated. We thus firstly performed the MeRIP-qPCR assay, showing that PHF1 mRNA contained abundant m6A modifications in A549 and PC-9 cells (Fig. 3A). Then, we compared and evaluated the potential associations between PHF1 and well-known demethylases or methyltransferases based on TCGA-LUAD cohort (https://portal.gdc.cancer.gov/). Intriguingly, PHF1 expression exhibited the most positive correlation with FTO, as compared with other m6A modification process-related proteins (Fig. 3B). Besides, we constructed stable FTO overexpression or knockdown cells using lentiviruses to assess the roles of FTO in LUAD. Of note, enhanced FTO expression levels could significantly elevate PHF1 levels, whereas FTO knockdown resulted in reduced PHF1 proteins (Fig. 3C). Accordingly, we transfected A549 cells with FLAG-FTO to overexpress FTO proteins and RNA immunoprecipitation suggested that PHF1 pre-mRNA could efficiently interact with FLAG-FTO (Fig. 3D). In addition, FTO knockdown could expectedly elevate PHF1 pre-mRNA containing m6A, as indicated by the methylated RNA immunoprecipitation–qPCR assay (Fig. 3E). However, only wild-type FTO, but not the catalytically inactive mutant (R316Q), could completely reverse this increase, implicating that PHF1 mRNA was regulated by FTO-dependent m6A demethylation (Fig. 3E). Given that m6A modification on transcripts mainly contributes to stability and degradation of mRNA, we found that PHF1 mRNA was destabilized when RNA synthesis was suppressed by actinomycin D in FTO-knockdown A549 cells (Fig. 3F). Particularly, the impaired PHF1 mRNA stability could only be restored by overexpressing wild-type FTO, but not the defective R316Q mutant in FTO-KD cells (Fig. 3F). As is well documented, YT521-B homology domain family (YTHDF) members are the readers to recognize m6A and contribute to degradation of m6A-containing RNAs. We therefore investigated the functions of YTHDF family proteins (YTHDF1, YTHDF2, and YTHDF3) to explore the negative relationships between m6A methylation and PHF1 mRNA stability. We observed that only YTHDF2 could efficiently interact with PHF1 pre-mRNA upon FTO knockdown, as revealed by the RNA immunoprecipitation–qPCR assay (Fig. 3G–H). Lastly, YTHDF2 depletion by specific siRNA indeed attenuated the effects of FTO knockdown on PHF1 mRNA levels, suggesting that FTO deficiency depends on YTHDF2 to trigger the degradation of PHF1 mRNA (Fig. 3I–K). Collectively, we highlighted that FTO-mediated m6A demethylation prohibited YTHDF2 from degrading PHF1 transcripts.

### Down-regulated FTO predicts poor prognosis of LUAD that has clinical significance

**Fig. 4:**
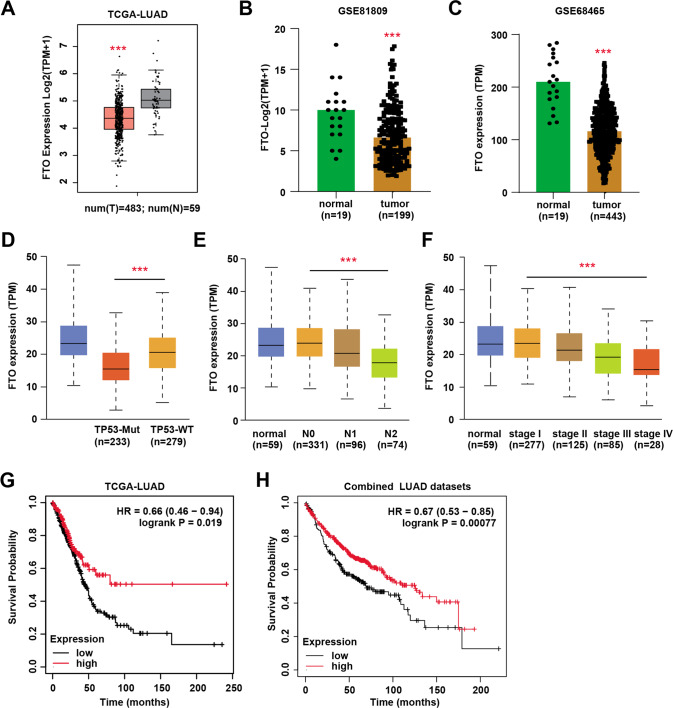
FTO was down-regulated in LUAD samples and associated with clinical significance in LUAD patients. **A** Box plot showing the differential FTO mRNA levels in normal and tumor samples. **B** Differential analysis of FTO levels in normal and tumor samples based on GSE81809 dataset. **C** Differential analysis of FTO levels in normal and tumor samples based on GSE68465 dataset. **D–F** Correlation analysis showing the underlying relationships between FTO levels and clinical phenotypes, including TP53-mutation status (**D**), lymphatic metastasis (**E**) and clinicalpathological stages (**F**). **G–H** Kaplan–Meier analysis with log-rank test showed that patients with low FTO had poorer OS outcomes relative to those with high FTO expressions based on TCGA-LUAD cohort (**G**) and combined pan-LUAD datasets (**H**). Bar = Mean ± SD. *P < 0.05, **P < 0.01, ***P < 0.001.

Previous studies have already elucidated the essential roles of FTO in regulating progression of multiple cancers, but its relationship with LUAD is still indefinite. We analyzed the expression data of FTO in LUAD samples and found that FTO was also down-regulated in tumor samples than normal tissues, which were demonstrated in other two independent datasets incorporating GSE81809 (*N* = 199) and GSE68465 (*N* = 443) (Fig. 4A–C). In addition, down-regulated FTO levels correlated with TP53-mutation status, N stages and advanced clinicalpathological stages (Fig. 4D–F). Lastly, we also divided the LUAD samples into FTO-low and FTO-high groups to perform the survival analysis. As implicated by TCGA-LUAD cohort and combined pan-LUAD datasets, patients with low FTO levels suffered from poorer OS outcomes than those in FTO-high group (Fig. 4G–H). Taken together, in line with PHF1, these data indicated that FTO also expressed lowly in LUAD patients and predicted poor prognosis.

### PHF1 overexpression restricted tumor growth induced by FTO knockdown in vitro and in vivo

**Fig. 5:**
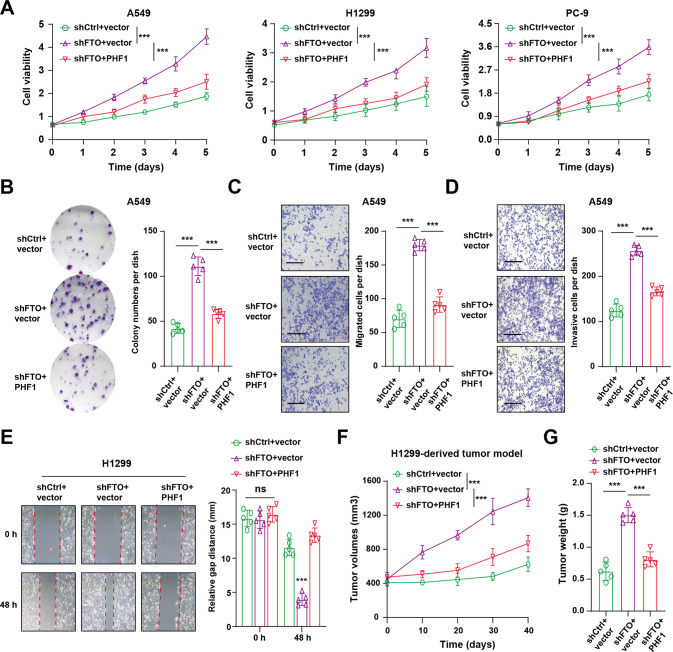
PHF1 overexpression restored the elevated tumor progression induced by FTO inhibition. **A** CCK-8 assays showing the in vitro cell growth capacity of FTO-KD cells transfected with vector or PHF1 in three LUAD cell lines (A549, H1299 and PC-9) (*N* = 5 per group). **B** Colony formation assay was conducted in FTO-KD A549 cells transfected with vector or PHF1. **C** Migration assay was conducted in FTO-KD A549 cells transfected with vector or PHF1. **D** Invasive assay was carried out in FTO-KD A549 cells transfected with vector or PHF1. **E** Wound-Healing assay was conducted in FTO-KD A549 cells transfected with vector or PHF1. **F** Quantification of tumor volumes in the H1299-derived tumor model incoporating three groups: shCtrl+vector, shFTO+vector and shFTO+PHF1. **G** Quantification of tumor weight in the H1299-derived tumor model incoporating the above three groups. Bar = Mean ± SD. *P < 0.05, **P < 0.01, ***P < 0.001.

Considering the clinical significance of FTO and FTO-mediated PHF1 regulation in LUAD samples, we further intended to figure out its biological roles. Firstly, we found that FTO knockdown could notably enhance cell growth, indicating that FTO may be tumor suppressor in LUAD. Ectopic expression of PHF1 in FTO-deficient cells could significantly restore the cell growth capacity. Besides, we also observed that targeting FTO could result in an increase of cell colony formation ability (Fig. 5B), cell migration (Fig. 5C) and invasion abilities (Fig. 5D–E), as compared to control cells. However, overexpression of PHF1 in these cells could all suppress the increase (Fig. 5B–E). To further investigate the in vivo roles of FTO/PHF1 axis in LUAD, we utilized the modified H1299 cells to perform the subcutaneous tumor model incorporating three groups (shCtrl+vector, shFTO+vector, shFTO+PHF1). In line with the in vitro findings, FTO inhibition induced growth rates of tumors relative to control tumors, whereas PHF1 overexpression could attenuate the elevated growth, as quantified and compared by the tumor volumes and tumor weight (Fig. 5F–G). In conclusion, these data supported that FTO exerted the anti-tumor effect and PHF1 overexpression could reverse the tumor progression of FTO-deficient LUAD in vitro and in vivo.

### Down-regulated FTO/PHF1 axis triggers FOXM1 accumulation to sustain stemness features and tumor progression

**Fig. 6:**
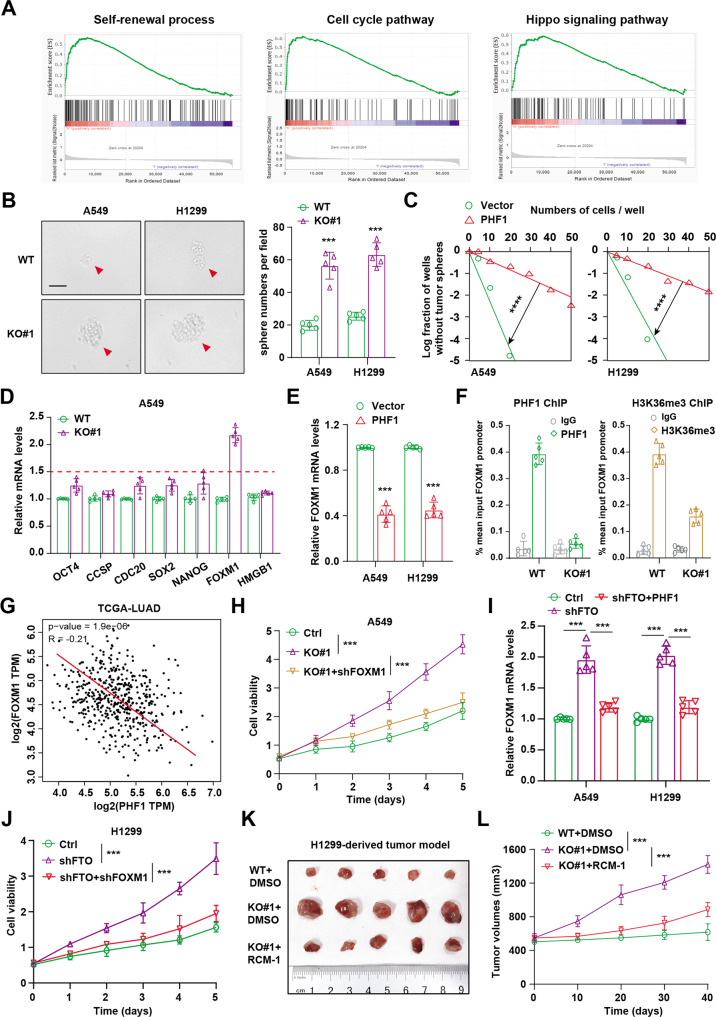
Down-regulated FTO/PHF1 axis enhanced the tumor self-renewal capacity and tumor progression in vitro and in vivo. **A** Gene set enrichment analysis (GSEA) was conducted to compare the enriched pathways in PHF1-low and PHF1-high groups. **B** Tumorsphere images of LUAD CSCs in parental WT and PHF1-KO groups (Scale bar = 200 μm). Quantification was shown on the right. **C** In vitro limiting dilution analysis of the tumorsphere formations of LUAD CSCs (WT & KO#1). PHF1 deficiency enhanced the self-renewal capacity of LUAD CSCs. **D** The qPCR assay screening the representative stemness-associated signature in parental and PHF1-KO A549 cells. **E** The qPCR assay detecting the FOXM1 mRNA levels in LUAD cells (A549, H1299) transfected with vetctor and PHF1. **F** ChIP-qPCR assays were performed to detect the enrichment of PHF1 and corresponding H3K36me3 at the promoter loci of FOXM1. **G** Gene correlation analysis was conducted with Spearson statistics between PHF1 and FOXM1 based on the TCGA-LUAD cohort. **H** CCK-8 assay was conducted to detect the in vitro cell growth in PHF1-deficient cells infected with shCtrl or shFOXM1. **I** The qPCR assay was conducted to detect the relative FOXM1 mRNA levels in FTO-KD cells transfected with vector or PHF1. **J** CCK-8 assay was performed to detect the in vitro cell growth in FTO-KD cells infected with shCtrl or shFOXM1. **K** Representative images of mice with xenografts tumors derived from H1299 cells. **L** Quantification of tumor volumes at indicated timepoints in the above subcutaneous model. Bar = Mean ± SD. *P < 0.05, **P < 0.01, ***P < 0.001.

Considering that down-regulated FTO contributed to low PHF1 levels to drive progression of LUAD, we thus intended to explore the potential mechanisms underlying the deficient FTO/PHF1 axis. First of all, we utilized the expression matrix of LUAD samples to conduct gene set enrichment analysis (GSEA) between PHF1-high and PHF1-low groups. The bioinformatic analysis implicated several oncogenic pathways that were significantly enriched in PHF1-low samples, including self-renewal pathway, cell cycle crosstalk and hippo signaling pathway (Fig. 6A). Given that intensive documents revealed that stemness features robustly contribute to tumor progression and drug resistance of LUAD, we intended to explore the relationships between PHF1 and tumor stemness capacity. Besides, PHF1 deficiency notably promoted cell stemness ability as evaluated by tumorsphere formation assays (Fig. 6B) and in vitro limiting dilution assays, in line with the above bioinformatic results (Fig. 6C). In line with this finding, we also found FTO knockdown could notably enhance cell stemness ability, whereas ectopic expression of PHF1 could suppress the self-renewal capacity of LUAD cells (Fig. S1A). Then, we screened and detected the representative stemness-related signature, finding that only FOXM1 showed the most increase in PHF1-deficient cells compared with control cells (Fig. 6D). In contrast, PHF1 overexpression could suppress FOXM1 mRNA levels in A549 and H1299 cells (Fig. 6E). Independent ChIP-qPCR experiments confirmed that PHF1 was present at FOXM1 loci, consistent with its epigenetic role (Fig. 6F). Previous studies have already indicated that PHF1 can mediate deposition of the repressive H3K36me3 mark at target genes, and we further found that PHF1 loss markedly reduced H3K36me3 levels at the FOXM1 gene loci (Fig. 6F). Given that PHF1 accompanies with H3K27me3 modification to restrict FOXM1 expressions, we further validated the negative relationships between PHF1 and FOXM1 in TCGA-LUAD samples (Fig. 6G). Targeting FOXM1 could effectively suppress the cell growth of PHF1-deficient A549 cells (Fig. 6H). Considering the identified regulations of FTO on PHF1 levels, we further found that FTO knockdown could also elevate FOXM1 levels, whereas ectopic PHF1 overexpression could reverse the increase (Fig. 6I and Fig. S1B). Meanwhile, FOXM1 inhibition could also effectively impair the cell growth of FTO-knockdown H1299 cells (Fig. 6J). Lastly, we utilized the H1299 cells to establish subcutaneous tumor model and found that FOXM1 inhibitor, RCM-1, could significantly suppress the in vivo growth of tumors derived from the PHF1-deficient cells, as evidenced and compared by tumor volumes (Fig. 6K–L). Taken together, our findings indicated that down-regulated FTO/PHF1 axis triggered the tumor stemness features to drive progression by activating FOXM1. Targeting FOXM1 using specific inhibitor (RCM-1) could significantly suppress malignant features of PHF1-deficient tumors, highlighting the clinical translational significance in LUAD treatment.

## Discussion

Aberrant interplay between N6-methyladenosine (m6A) modification and epigenetic chromatin proteins represents the biological hallmark that contributes to multiple tumors [24, 25]. From one aspect, m6A modification could regulate the stabilities of epigenetic regulators to trigger chromatin remodeling and tumor progression. Yunhao Chen et al. have found that WTAP could rely on m6A modification to mediate the posttranscriptional suppression of ETS proto-oncogene 1 (ETS1) in liver cancer [26]. Besides, METTL14-mediated m6A modification negatively modulated the mRNA stability of bromodomain PHD finger transcription factor (BPTF), which could remodel the enhancer landscape to reinforce RCC proliferation [27]. Moreover, METTL3 could epigenetically suppress the YPEL5 in an m6A-YTHDF2-dependent manner by regulating the m6A site in the coding sequence region of the YPEL5 transcript [28]. From another aspect, LNC942 directly recruits METTL14 protein by harboring the specific recognize sequences, thereby stabilizing the expression of downstream targets of LNC942 including CXCR4 and CYP1B1 through posttranscriptional m6A methylation modification [29]. Meanwhile, protein arginine methyltransferase 1 (PRMT1) interacts with, and methylates the intrinsically disordered C terminus of METTL14, which enhancing its RNA methylation activity in DNA repair process [30]. As a result, efforts focusing on regulations between m6A and epigenetic remodeling events are warranted to facilitate our better understanding of LUAD tumorigenesis. In the current study, we utilized the bioinformatic analysis to find that PHF1 expressed lowly in LUAD than normal lung tissues. We demonstrated our findings in other datasets and collected samples. Besides, we also observed that PHF1 correlated negatively with clinical characteristics. Prognostic analysis further revealed that patients with low PHF1 had poorer survival outcomes relative to those with high PHF1 levels. These data supported the idea that PHF1 may be a tumor suppressor in LUAD. Subsequently, we conducted the experimental assays and proved that PHF1 inhibition could result in an increase of cell proliferation and migration in vitro, while ectopic expression of PHF1 could restore the effect. Targeting PHF1 also enhanced the in vivo orthotopic lung tumor growth. Mechanistically, we analyzed that FTO correlated positively with PHF1 expression. FTO was found to interact with PHF1 pre-mRNA and stabilize its levels via demethylating m6A modification. We further elucidated that FTO-mediated m6A demethylation prevented YTHDF2 from degrading PHF1 transcripts. FTO also expressed lowly in LUAD that correlated with poor prognosis. Targeting FTO could promote LUAD progression and ectopic expression of PHF1 restored the effect. Lastly, we also found PHF1 could coordinate with H3K36me3 to restrict FXOM1 expressions, thus down-regulated FTO/PHF1 axis triggered the elevated FXOM1 levels and promoted the self-renewal capacity of LUAD. We proposed that targeting FOXM1, using specific inhibitor (RCM-1), could inhibit the growth of PHF1-deficient LUAD, creating a treatment vulnerability for clinical utility.

Given that intensive studies have indicated FTO may exert opposite functions in different tumors, its roles with LUAD progression still remain unclear. However, our study and results supported that FTO mainly had the anti-tumor activity in LUAD. Decreased FTO levels may predict the poorer prognosis of LUAD and has clinical significance. Similarly, Dan-Yun Ruan et al. also demonstrated that reduced FTO protein expression was associated with a high recurrence risk and poor prognosis in resectable colon cancer patients. FTO mainly played a tumor-suppressive role via reducing metastasis-associated protein 1 (MTA1) expression in an m6A-dependent manner [31]. As reported, lots of mechanisms could regulate the expressions and activity of FTO. FTO is a member of the Fe-II and 2-oxoglutarate-dependent dioxygenase superfamily [32]. As an oncometabolite, R-2-hydroxyglutarate (R-2HG) could sustain relatively high levels by mutant isocitrate dehydrogenase 1/2 (IDH1/2) enzymes [33]. Mechanistically, R-2HG suppressed FTO activity and elevated the global m6A modification levels in R-2HG-sensitive leukemia cells. Besides, SIRT1 activated RANBP2, indispensable for FTO SUMOylation at Lysine (K)−216 site, to enhance FTO degradation, leading to progression of hepatocellular carcinoma [34]. Although we have observed that FTO expressed lowly in LUAD, we were still uncertain about the up-stream regulatory mechanisms of FTO that contribute to its low levels.

A better understanding of cancer stem cell biology in lung cancer is essential to develop effective therapies. In this study, we confirmed that decreased expressions of FTO/PHF1 axis could induce enhanced self-renewal capacity of LUAD CSCs. Previous studies have found the opposite results, where genetic depletion or pharmacological inhibition of FTO could suppress leukemia stem/initiating cell self-renewal and reprogram immune response [35]. Based on the low-throughput screening of stemness-related signature, we detected that PHF1 mainly inhibited FOXM1 mRNA levels. FOXM1 is found to be a pro-oncogene transcription factor contributing greatly to cell cycle progression. FXOM1 linked closely with the expression levels of stem cell markers (Nanog, Sox2, and OCT4) in various tumor samples, and also in turn potentiated the expression of these stemness-related genes in vitro [36]. In addition, FOXM1 upregulation stimulates the Wnt/β-catenin signaling crosstalk by directly binding to β-catenin, thereby preserving self-renewal ability of leukemia CSCs [37]. The researchers have previously discovered the small-molecule compound, Robert Costa Memorial drug-1 (RCM-1) for particularly inhibiting FOXM1 [38]. RCM-1 decreased FOXM1 protein in the tumors, reduced tumor cell growth, and increased tumor cell apoptosis. Of note, RCM-1 could also inhibit protein levels and nuclear localization of β-catenin, abolishing interactions between β-catenin and FOXM1 in cultured tumor cells and in vivo. Based on the above fundamental basis, we found RCM-1 could largely inhibit the in vivo growth of PHF1-deficient LUAD. We further speculated that RCM-1 might be more suitable for PHF1^low/−^ tumors, but not the PHF1^high^ cases, which needs further validations and more pre-clinical models to prove.

This study still has some limitations. First of all, we are still uncertain about the optimal cutoff to stratify PHF1^low^ and PHF1^high^ LUAD samples. Using immunohistochemistry (IHC) data to define low PHF1 expression in LUAD may be somehow arbitrary, and eventual clinical use requires large samples to eliminate errors. Secondly, apart from FTO, whether other m6A-related enzymes, like METTL3/14, ALKBH5, could regulate PHF1 expressions remain unclear. Last but not least, clinical implications of RCM-1 need to be further investigated, and patient derived tumor xenograft may be recommended to make the results more solid. In addition, we observed that low FTO expressions correlated with TP53 mutations, implicating the associations between TP53 and FTO levels. Whether TP53 status could contribute to down-regulated FTO expressions remains to be further validated in LUAD.

## Conclusion

In summary, we uncovered the biological roles of PHF1 in LUAD. Down-regulated FTO failed to prohibit YTHDF2 from degrading PHF1 transcripts, thereby suppressing PHF1 levels. Decreased FTO/PHF1 levels correlated with poorer prognosis of LUAD and predicted unfavorable outcomes. Finally, we also found that decreased FTO/PHF1 axis potentiated FOXM1 levels to enhance self-renewal capacity of LUAD. Targeting FOXM1 may be a therapeutical vulnerability in PHF1^low/−^ LUAD, which possessed remarkable clinical significance.

## Methods and materials

### Cell lines, antibodies and chemicals

All LUAD cell lines (A549, H1299, PC-9) and 293T cells were obtained from ATCC (American Type Culture Collection, Rockville, MD, USA). Then, these cells were maintained in DMEM/DME/F-12 1:1 (Dulbecco’s modified Eagle’s medium/Ham’s nutrient mixture F-12) (Invitrogen), added with 10% fetal bovine serum (FBS) and 5% penicillin. All cell lines were cultured in a humidified atmosphere under 37 °C and 5% CO_2_. We obtained the growth media, reagents, and supplements from Gibco. PHF1 antibody (ab184951), FTO antibody (ab126605) and β-actin antibody (ab8226) were all obtained from Abcam. All antibodies were diluted according to the instructions. The RCM-1 (FOXM1 inhibitor) was obtained from the MCE company.

### Transfection and infection, PHF1-KO construction

FTO-specific shRNAs and control shRNAs were purchased from Addgene and inserted into the pLKO.1 vector. The sequences were listed as following: shFTO#1: GTCTCGTTGAAATCCTTTGAT; shFTO#2: CCAGGGAGACTGCTATTTCAT. The plasmid encoding human PHF1, was established by PCR-based amplification and subsequently subcloned into the pCDH-CMVMCS-EF1-copGFP vector. The 293T cell lines were co-transfected with the packaging plasmids and the corresponding shRNA plasmids or overexpressing plasmids to generate lentivirus. Lipofectamine 2000 was utilized for all transfections according to the manufacturer’s instructions. At 48 h after transfection, the virus supernatant was collected to infect LUAD cells, supplemented with 4 μg/mL polybrene. For the knockout assay, we utilized the pX459 plasmids to clone sgRNA guide oligos that delete PHF1. The designed sgRNAs were listed as following: PHF1-sgRNA#1: F: 5′-CACCGTGTCTTTGCGATCGCCACCA-3′; R: 5′-AAACTGGTGGCGATCGCAAAGACAC-3′. PHF1-sgRNA#2: F: 5′-CACCGTGGCGATCGCAAAGACACAC-3′; R: 5′-AAACGTGTGTCTTTGCGATCGCCAC-3′. A549 and PC-9 cells were plated and transfected with pX459 constructs for 24 h. Then, 1 μg/ml puromycin was used to kill cells for 3 days. The left LUAD cells were digested and seeded in 96-well plate to get monoclonal cell line. Western blot was used to screen effective PHF1-KO cell lines.

### Cell proliferation assay

The MTT experimental assay was utilized to generate cell growth curves. Briefly, 1 × 10^3^ A549 or PC-9 cells were seeded in flatbottomed 96-well plates for 6 days to assess cell viability. From Day 0 to Day 6, the MTT assays were conducted in line with the manufacturer’s instructions. All experiments were repeated three times independently.

### Transwell migration and invasion assay

A549 and PC-9 cells were seeded in the upper chambers of a 24-well transwell (Corning, Beijing, China). The membranes were painted with Matrigel in the invasion assay (BD Biosciences). Cells with serum-free media were added to the upper chamber. The lower chambers were filled with culture medium supplemented with 10% FBS. The A549 or PC-9 cells were fixed in 4% paraformaldehyde for 15 min and then incubated with crystal violet after 48 h incubation. The acounts of cells were assessed from five randomly selected microscopic fields per filter and observed by the brightfield microscopy.

### Wound-healing assay

H1299 cells were cultured for 48 h to reach 80% confluency, and then a straight artificial wound was scraped with a 200 μl pipette tip. The cell migration ability was measured by photographing the distance at 0 and 24 h.

### Western blot

The lysis buffer was prepared that contains 50 mM TrisHCl, pH 7.5, 150 mM NaCl, 1% Nonidet P-40, 0.25% sodium deoxycholate, 0.1% sodium dodecyl sulfate (SDS) with a complete protease inhibitor cocktail (Roche) and phosphatase inhibitors (Sigma-Aldrich). The SDS–polyacrylamide gel electrophoresis (SDS-PAGE) was used to separate cell lysates. After blocked by 5% milk for 1 h, the membrane bands were incubated by primary antibody and the subsequent secondary antibody.

### Immunohistochemistry

The immunological tissue used in the experiment was purchased from Alenabio. Paraffin-embedded tumor LUAD specimens were cut into 5 mm slices and then deparaffinized and rehydrated. Totally, 50 paired tumor samples and normal tissues were obtained. Each LUAD sample did not receive any chemotherapy or other treatment at the first diagnosis. The sections were immersed in 10 mM citrate buffer (pH = 6.0) and heated in a microwave oven for 20 min for antigen repair. After quenching endogenous peroxidase activity and blocking with normal goat serum, the slices were incubated with primary antibodies (PHF1) followed by horseradish peroxidase-linked secondary antibody. Slices were visualized with (diaminobenzidine) DBA and counterstained with hematoxylin before observing with a microscope. Informed consent was obtained from each LUAD patient or their guardians, and the study were approved by the Ethics Committee of the Harbin Medical University Cancer Hospital.

### RNA-binding protein immunoprecipitation (RIP) and m6A RNA immunoprecipitation (MeRIP) assay

RIP assays were conducted with a Magna RIPTM RNA-Binding Protein Immunoprecipitation Kit (Millipore) according to the manufacturer’s protocol. Briefly, the cells were collected and lysed in a complete radioimmunoprecipitation assay buffer containing a protease inhibitor (cocktail) and RNase inhibitor. Antibodies (5 μg) were pre-bound to Protein A/G magnetic beads in immunoprecipitation buffer (20 mM Tris-HCl pH 7.5, 140 mM NaCl, 0.05% TritonX-100) for 2 h and then incubated with 100 μL of cell lysate overnight at 4 °C with rotation. RNA was eluted from the beads by incubation with 400 μL of elution buffer for 2 h, precipitated with ethanol, and dissolved in RNase-free water. The enrichment of certain fragments was determined by real-time PCR. MeRIP assays were performed using a Magna MeRIP

m6A Kit (Millipore) according to the manufacturer’s instructions. Briefly, RNAs were chemically fragmented to ~100 nucleotides and incubated with magnetic beads conjugated to m6A antibodies (Abcam) for immunoprecipitation. The enrichment of m6A-containing mRNA was analyzed by qRT-PCR and normalized to the input.

### Chromatin immunoprecipitation assays

LUAD cells were crosslinked with 1% formaldehyde and quenched in 0.125 M glycine. The 1.5 × 10^7^ A549 cells were crosslinked by 1% formaldehyde for 10 min at room temperature, following by 5 min blocking with 0.25 M glycine. The pellets were lysed and chromatin was sheared into 500–1000 bp by sonication. The chromatin fragments were incubated with indicated antibody and protein A/G agarose beads at 4 °C overnight. After wash and reverse-crosslink, the purified DNA was quantified by qPCR with specific primers.

### Tumorsphere formation assay

A549 or PC-9 cells were suspended and seeded into six-well ultralow attachment plates with 3 ml/well cancer stemness medium containing 2xB27, 1xN2 supplement, 20 ng/ml EGF (epidermal growth factor) and 10 ng/ml bFGF (basic fibroblast growth factor). After cultivation for 2 weeks, tumorsphere formation was counted and analyzed by microscopy in five random fields. The experiment was executed in triplicate.

### Animal assay

A total of 40 4–6 weeks BALB/c nude mice were purchased from Shanghai Lingchang Biotechnology Co., Ltd. and housed in a specific pathogen-free environment. The animal assays comlied with the blinding role. The mice were randomly divided into groups before subcutaneous injection with 1 × 10^7^ cells that were suspended in 150 μL of phosphate-buffered saline. Tumors were measured at indicated timepoints after injection. Tumor volume was calculated using the following formula: V (mm3) = 0.5 × length (mm) × width2 (mm2). For the orthotopic lung cancer model, the uciferase-tagged A549 cells (parental, PHF1-KO#1) were obtained by transfection of luciferase reporter gene and selection procedure were orthotopically injected through pleural (1 × 10^6^ cells in media with Matrigel, 1:1 ratio in volume) into 8 weeks nude mice. Tumor growth was monitored once a week by in vivo Image System with luciferin injection.

### Bioinformatic analysis

Differential analysis was used by the limma package. Correlation analysis was conducted to determine the relationships between FTO/PHF1 expressions and clinical factors. Kaplan–Meier analysis was conducted by the survival package. Gene Set Enrichment Analysis (GSEA) was conducted to figure out the related pathways downstream of PHF1. Hallmark and C7 gene sets v6.2 collections were downloaded from Molecular Signatures Database as the target sets. The transcriptome data of all TCGA-LUAD samples was utilized for GSEA, and only gene sets with *p* < 0.05 and FDR *q* < 0.05 were considered as significant.

### Statistical analysis

A two-tailed Student’s *t* test was used to calculate the significance. The correlation between genes and clinical factors were determined using Pearson analysis. All data were expressed as the Mean ± SD. All experiments were carried out in triplicate, and the *P* < 0.05 was considered to be statistically significant. The SPSS 22.0 and R studio (Version 3.5.1) were used for the statistical analyses.

## Supplementary information

Supplementary Figures

Table S1

Original Data File

Raw statistical data

## Data Availability

The data used to support the fndings of this study are available from the corresponding author upon request. TCGA-LUAD data was obtained from the GDC portal (https://portal.gdc.cancer.gov/).
