# Supplementary material for: Down-regulated m6A reader FTO destabilizes PHF1 that triggers enhanced stemness capacity and tumor progression in lung adenocarcinoma
Source: Cell Death Discov. 2022 Aug 9;8:354. doi: 10.1038/s41420-022-01125-y (PMC9363432; doi:10.1038/s41420-022-01125-y)
Supplement: Supplementary file 1 — Supplementary Figures [file 41420_2022_1125_MOESM1_ESM.docx]

**
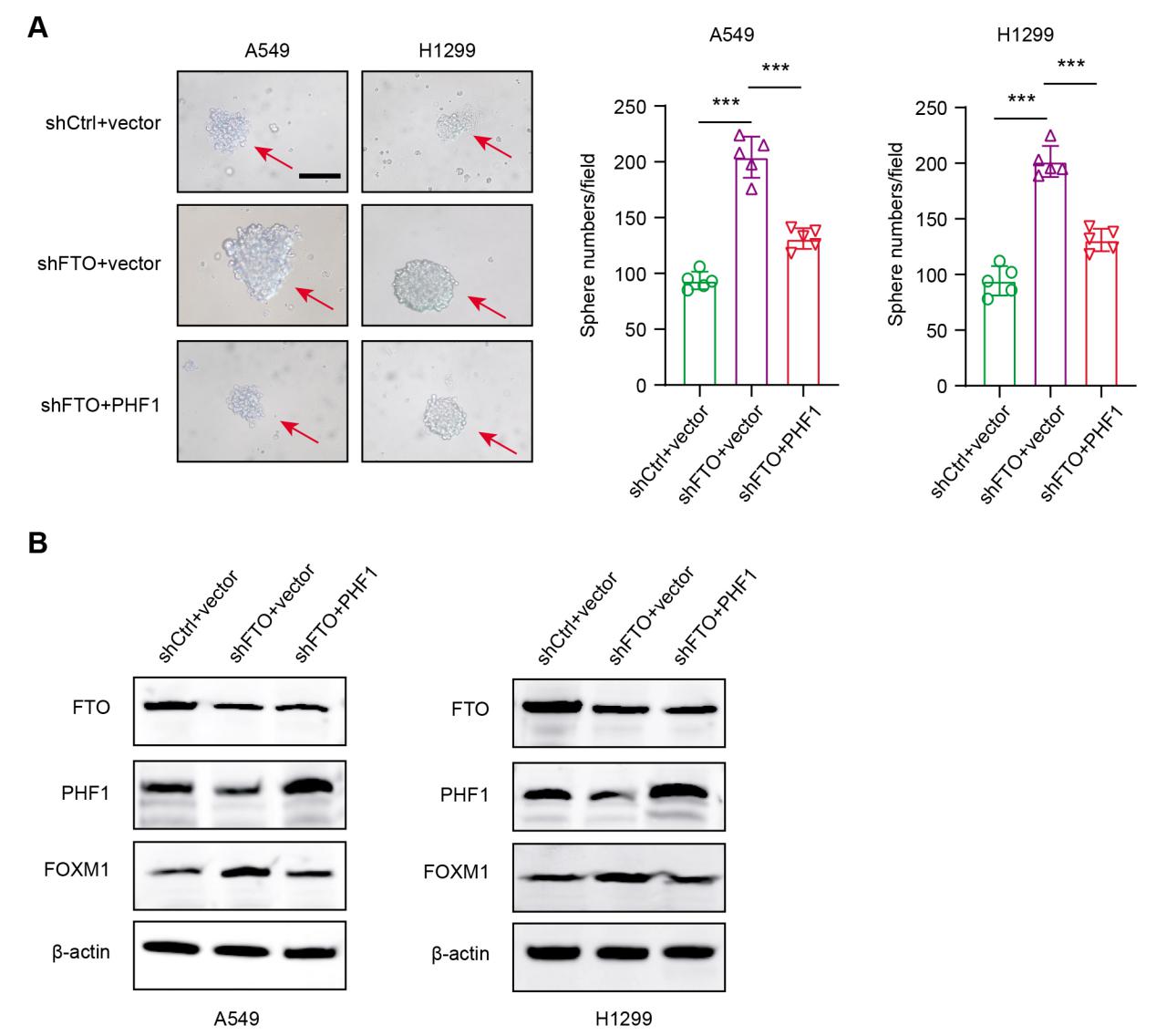
**

**Figure S1 FTO/PHF1 axis regulates tumor sphere formation abilities via FOXM1. (A)** FTO knockdown could enhance tumor self-renewal abilities, while PHF1 overexpression could suppress the capacties in A549 and H1299 cells. Scale bar = 200 μm. **(B)** FTO knockdown could enhance FOXM1 expressions, while PHF1 overexpression could suppress the levels of FOXM1 in A549 and H1299 cells.
