# Supplementary figures and images for: Down-regulated m6A reader FTO destabilizes PHF1 that triggers enhanced stemness capacity and tumor progression in lung adenocarcinoma
Source: Cell Death Discov. 2022 Aug 9;8:354. doi: 10.1038/s41420-022-01125-y (PMC9363432; doi:10.1038/s41420-022-01125-y)

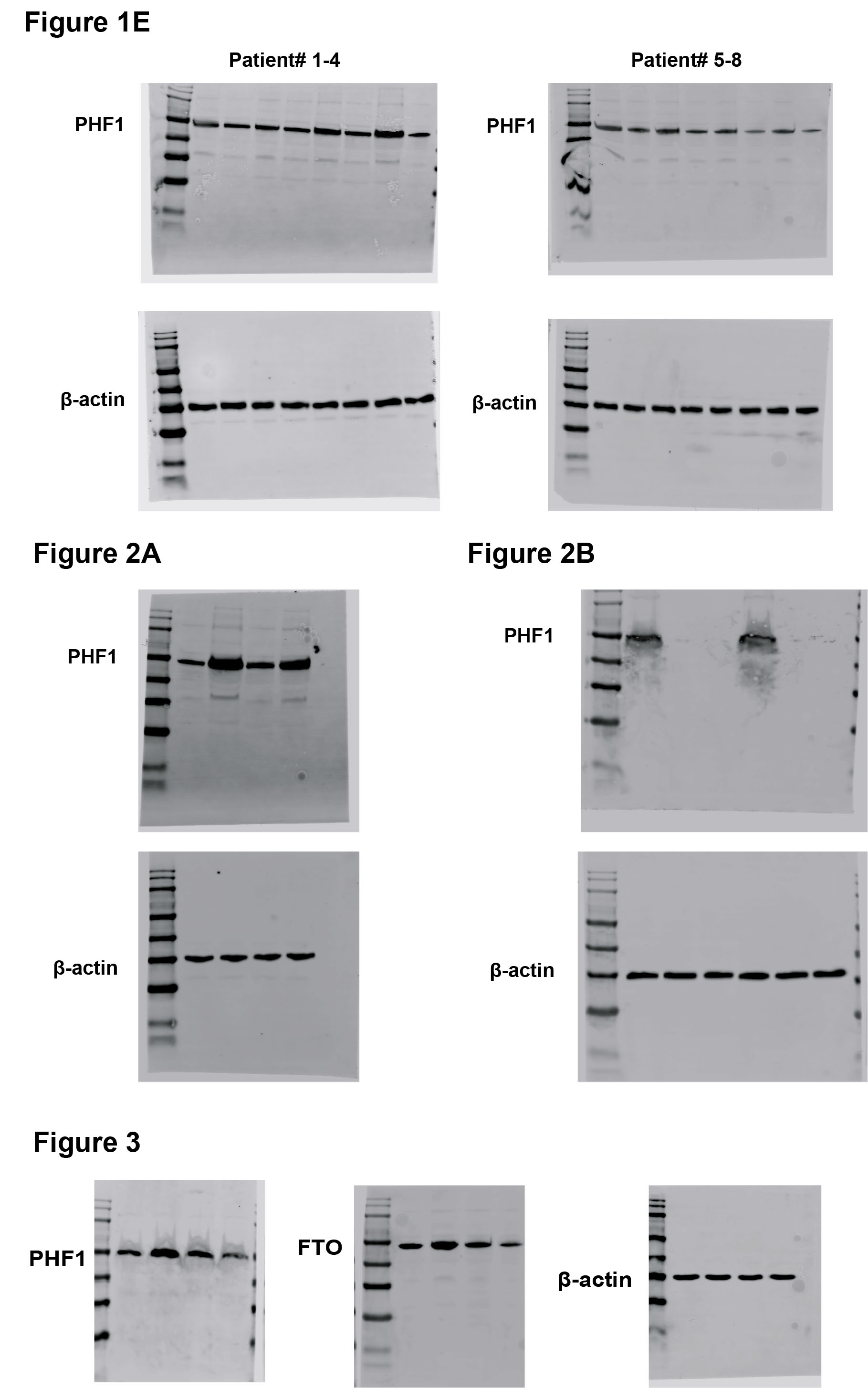

Supplement: Supplementary file 3 — Original Data File [file 41420_2022_1125_MOESM3_ESM.jpg]
